# Advances in Completely Automated Vowel Analysis for Sociophonetics: Using End-to-End Speech Recognition Systems With DARLA

**DOI:** 10.3389/frai.2021.662097

**Published:** 2021-09-24

**Authors:** Rolando Coto-Solano, James N. Stanford, Sravana K. Reddy

**Affiliations:** Dartmouth College, Hanover, NH, United States

**Keywords:** sociophonetics, vowels, dialects, American English, automated speech recognition, linguistics, Northern cities vowel shift, Southern vowel shift

## Abstract

In recent decades, computational approaches to sociophonetic vowel analysis have been steadily increasing, and sociolinguists now frequently use semi-automated systems for phonetic alignment and vowel formant extraction, including FAVE (Forced Alignment and Vowel Extraction, Rosenfelder et al., 2011; Evanini et al., Proceedings of Interspeech, 2009), Penn Aligner (Yuan and Liberman, J. Acoust. Soc. America, 2008, 123, 3878), and DARLA (Dartmouth Linguistic Automation), (Reddy and Stanford, DARLA Dartmouth Linguistic Automation: Online Tools for Linguistic Research, 2015a). Yet these systems still have a major bottleneck: manual transcription. For most modern sociolinguistic vowel alignment and formant extraction, researchers must first create manual transcriptions. This human step is painstaking, time-consuming, and resource intensive. If this manual step could be replaced with completely automated methods, sociolinguists could potentially tap into vast datasets that have previously been unexplored, including legacy recordings that are underutilized due to lack of transcriptions. Moreover, if sociolinguists could quickly and accurately extract phonetic information from the millions of hours of new audio content posted on the Internet every day, a virtual ocean of speech from newly created podcasts, videos, live-streams, and other audio content would now inform research. How close are the current technological tools to achieving such groundbreaking changes for sociolinguistics? Prior work (Reddy et al., Proceedings of the North American Association for Computational Linguistics 2015 Conference, 2015b, 71–75) showed that an HMM-based Automated Speech Recognition system, trained with CMU Sphinx (Lamere et al., 2003), was accurate enough for DARLA to uncover evidence of the US Southern Vowel Shift without any human transcription. Even so, because that automatic speech recognition (ASR) system relied on a small training set, it produced numerous transcription errors. Six years have passed since that study, and since that time numerous end-to-end automatic speech recognition (ASR) algorithms have shown considerable improvement in transcription quality. One example of such a system is the RNN/CTC-based DeepSpeech from Mozilla (Hannun et al., 2014). (RNN stands for recurrent neural networks, the learning mechanism for DeepSpeech. CTC stands for connectionist temporal classification, the mechanism to merge phones into words). The present paper combines DeepSpeech with DARLA to push the technological envelope and determine how well contemporary ASR systems can perform in completely automated vowel analyses with sociolinguistic goals. Specifically, we used these techniques on audio recordings from 352 North American English speakers in the International Dialects of English Archive (IDEA^1^), extracting 88,500 tokens of vowels in stressed position from spontaneous, free speech passages. With this large dataset we conducted acoustic sociophonetic analyses of the Southern Vowel Shift and the Northern Cities Chain Shift in the North American IDEA speakers. We compared the results using three different sources of transcriptions: 1) IDEA’s manual transcriptions as the baseline “ground truth”, 2) the ASR built on CMU Sphinx used by Reddy et al. (Proceedings of the North American Association for Computational Linguistics 2015 Conference, 2015b, 71–75), and 3) the latest publicly available Mozilla DeepSpeech system. We input these three different transcriptions to DARLA, which automatically aligned and extracted the vowel formants from the 352 IDEA speakers. Our quantitative results show that newer ASR systems like DeepSpeech show considerable promise for sociolinguistic applications like DARLA. We found that DeepSpeech’s automated transcriptions had significantly fewer character error rates than those from the prior Sphinx system (from 46 to 35%). When we performed the sociolinguistic analysis of the extracted vowel formants from DARLA, we found that the automated transcriptions from DeepSpeech matched the results from the ground truth for the Southern Vowel Shift (SVS): five vowels showed a shift in both transcriptions, and two vowels didn’t show a shift in either transcription. The Northern Cities Shift (NCS) was more difficult to detect, but ground truth and DeepSpeech matched for four vowels: One of the vowels showed a clear shift, and three showed no shift in either transcription. Our study therefore shows how technology has made progress toward greater automation in vowel sociophonetics, while also showing what remains to be done. Our statistical modeling provides a quantified view of both the abilities and the limitations of a completely “hands-free” analysis of vowel shifts in a large dataset. Naturally, when comparing a completely automated system against a semi-automated system involving human manual work, there will always be a tradeoff between accuracy on the one hand versus speed and replicability on the other hand [Kendall and Joseph, Towards best practices in sociophonetics (with Marianna DiPaolo), 2014]. The amount of “noise” that can be tolerated for a given study will depend on the particular research goals and researchers’ preferences. Nonetheless, our study shows that, for certain large-scale applications and research goals, a completely automated approach using publicly available ASR can produce meaningful sociolinguistic results across large datasets, and these results can be generated quickly, efficiently, and with full replicability.

## Introduction

Phonetic alignment and extraction of vowel formants are central to modern sociophonetics (Thomas, 2011; Kendall and Fridland, 2021), and recent decades have seen a steady increase in automation for these important tasks. The FAVE system, Forced Alignment, and Vowel Extraction (Rosenfelder et al., 2011) provided one such semi-automated tool. With FAVE, users manually transcribe the text in Praat TextGrids (Boersma and Weenink 2019), upload to an automatic aligner (FAVE-Align), then use FAVE-Extract to extract the vowel formant frequencies. This produces an important improvement in processing time: Labov et al. (2013) report that, with 40 h of manual work his team could process the phonetic information of 300 vowels. On the other hand, using automatic alignment, up to 9,000 vowels could be processed in the same 40 h. But despite this progress, the current state-of-the-art methods still have to deal with an expensive and time-consuming bottleneck: the manual transcription of recordings. For accurate results, human transcribers must manually transcribe the audio. In this respect, most modern sociophonetic tools are “semi-automated,” in that they require human transcription (or at least human verification of a transcription) to then proceed to the automated extraction of phonetic information. This step of manual transcription takes an enormous amount of time and resources of human labor, and frequently introduces human error due to typographical errors or other problems during annotation.

The DARLA system, which is short for “Dartmouth Linguistic Automation” (darla.dartmouth.edu) (Reddy and Stanford (2015a-c), provides a user-friendly version of this workflow which has become prevalent in recent years with researchers and students around the world; over 25,000 jobs have been run on DARLA since 2015. DARLA has a web-based utility for simple uploads of transcriptions (TextGrids or plaintext) and audio. Unlike other systems, DARLA has both a semi-automated and a fully automated system for vowel alignment and extraction. Both systems use the Montreal Forced Aligner for the phonetic alignment (McAuliffe et al., 2017). In the semi-automated version, users manually transcribe the audio into either plaintext files or audio-aligned TextGrids. In the fully automated system, users upload audio and DARLA uses its own in-house automatic speech recognition system (ASR) to create a transcription. After the ASR process is complete, DARLA goes on to align and extract the vowel formants, matching the audio and transcription with the Montreal Forced Aligner and then extracting the formants using FAVE-Extract (Rosenfelder et al., 2011). DARLA’s current ASR is based on the CMU Sphinx toolkit, a HMM/GMM based ASR system. (HMM/GMM stands for “Hidden Markov Model, Gaussian Mixture Model,” the mechanism for finding the phones in the audio stream). Reddy and Stanford (2015c) show that DARLA’s fully automated transcription function can generate useful sociolinguistic results in a completely “hands-free” manner. The study used DARLA to automatically analyze US Southern and US Northern speakers, finding that the fully automated system could uncover statistically significant contrasts between the two regions in terms of the Southern Vowel Shift. Although these North-South contrasts were more clearly visible in the manually transcribed version, Reddy and Stanford (2015c) pointed out that despite limitations of the current ASR system, that fully automated system could still produce useful sociolinguistic results from some types of large-scale “big data” applications.

As examples a-d (reprinted from Reddy and Stanford, 2015c) below suggest, errors in the transcription may not affect the overall goal of producing vowel formants that are generally representative of a speaker’s dialect features. In these examples, the ASR system has made large errors in transcription which crucially affect the meaning of some of the sentences. But from the sociophonetician’s viewpoint, these errors may not affect the end result. In many cases, the extracted (stressed) vowel is the same for both systems, such as in the word those versus close and in spend versus depend. Naturally, phonetic environments may be affected [(z) in those versus (s) in close]. But for some large-scale applications, this may not be crucial. Reddy and Stanford (2015c) find that the US Southern Vowel Shift can be effectively diagnosed using such fully automated functions. Using 46 Southern and 47 Northern speakers in the Switchboard corpus (Godfrey and Holliman, 1993), they show a statistically significant difference between Southern speakers and Northern speakers, and they do this without needing any manual human transcribers.

a) *Manual*: give me your first impression. ASR: give me yours first impression
b) *Manual*: It’s one of those. ASR: It’s close
c) *Manual*: no It’s It’s wood turning. ASR: no it would turn it
d) *Manual*: and we really Don’t spend on anything. ASR: and we don’t depend on anything

Even though the fully automated pipeline has been shown to recover dialect differences, in practice, most users of DARLA depend on the semi-automated version with manual transcripts, since the word error rate of the CMU Sphinx ASR remains high.

In recent years, there have been numerous improvements in automatic speech recognition over the traditional HMM/GMM models. Two of them stand out: more availability of audio training data, and more powerful end-to-end deep learning algorithms. The amount of high-quality transcribed speech has exploded in recent years, and much of this data is available under open licenses. Two examples of such datasets are Mozilla’s crowd-sourced Common Voice (Ardila et al., 2019), which contains more than 1,600 h of English, where volunteers read elicited sentences, and the OpenASR’s LibriSpeech (Panayotov et al., 2015), which contains over 1,000 h of volunteers reading book passages. This has greatly increased the training data available to ASR algorithms, which themselves have improved during the last decade. The adoption of end-to-end algorithms has led to important reductions in transcription errors. These algorithms learn the word order and the phone acoustics together, rather than through separate language and acoustic models. They also build upon massive advances in deep learning, particularly in the capacity of their neural networks to understand the context of a word. The DeepSpeech algorithm (Hannun et al., 2014) uses these open corpora and combines them with an end-to-end architecture.

Our objective in this paper is to measure whether these end-to-end algorithms can provide transcriptions that are good enough to detect well-known sociophonetic patterns. There is research indicating that current ASR systems do not perform equally well with non-standard dialects of English (Tatman, 2017), so it is possible that such an experiment will fail to detect patterns such as the movements of vowels in the Southern dialect of US English. We conduct a test with speakers from all states and examine two regional dialects of US English: Southern and Inland North.

In the next sections we tackle the following questions: 1) Can automated transcriptions detect large-scale sociolinguistic patterns in a large dataset? 2) Can newer systems like DeepSpeech detect these patterns better than previous automated transcription methods? 3) How does an automated transcription fare against human-transcribed data in detecting sociolinguistic patterns? The first two questions will be studied in *Improvements in Sociophonetic Analysis* and *Variationist Analysis of NCS Movements*, and the third question will be studied in *Improvements in Sociophonetic Analysis* for the Southern Vowel Shift, and *Variationist Analysis of NCS Movements* for the Northern Cities Shift.

## Methods

**FIGURE 1:**
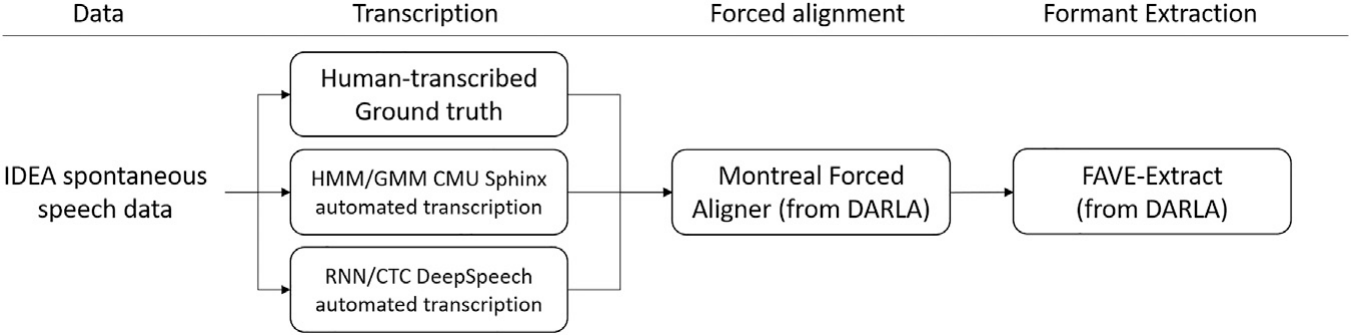
Workflow for data processing.

We produced three types of transcriptions: (i) the manually transcribed ground truth, based on the IDEA transcription but hand-corrected to ensure it matches the recordings (ii) an automated transcription produced by the DeepSpeech program, (iii) and an automated transcription using the previously existing DARLA system built upon CMU Sphinx (Lamere et al., 2003). DeepSpeech was used with a pre-trained model developed by Mozilla^2^, which is trained on the Fisher, LibriSpeech, Switchboard, and Common Voice English corpora in addition to 1700 h of transcribed NPR radio shows. The Sphinx system uses a model pretrained on a variety of American English speech corpora, mainly broadcast news and telephone conversations. Figure 1 shows a summary of the workflow for data extraction.

These transcriptions were produced for recordings of 352 speakers of American English included in the International Dialects of English Archive^3^ corpus (IDEA). All of the recordings are in a conversational, informal style, recorded in interviews asking the participants to talk about where they are from. There is only one recording per speaker, and, in total, the corpus contains approximately 12.5 h of audio (45,154 s). The recordings were an average of 128 ± 59 s long, with a minimum of 23 s and a maximum of 6 min 28 s. The corpus included 192 female and 160 male speakers, 54 and 46% respectively. The ages of the speakers at the time of recording ranged between 11 and 95 years old at the time of recording, with a median age of 37. The ethnic makeup of the sample is as follows: 79% was white (279), 10% was black (34), 5% was of Latin American descent (16), 3% was Native American (10), 0.28% was Asian American (1 person), 3% reported mixed ancestry 9), and 1% declared no ethnicity (3).

The data included speakers from every state in the United States. These speakers were grouped in three groups: Inland North, Southern, and General North. These three regional groupings made it possible for us to make regional comparisons of speakers in terms of the Southern Vowel Shift (SVS) and the Northern Cities Shift (NCS), as discussed below.

**FIGURE 2:**
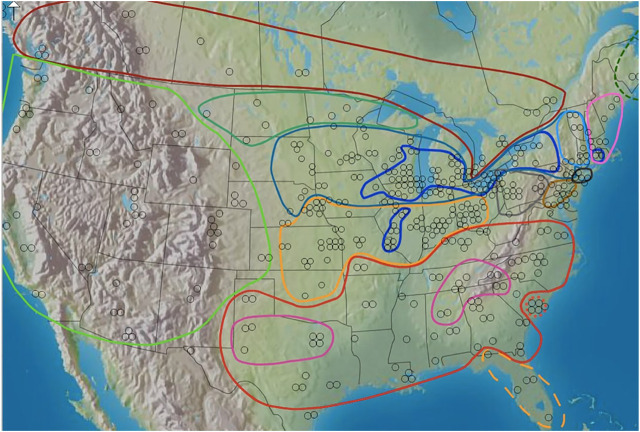
North American dialect regions as outlined in the ANAE. Dark blue = Inland North. Red = South. Map to be reprinted from Labov et al. (2006).

The Inland North group was defined according to the region identified as Inland North in the Atlas of North American English (ANAE) (Labov et al., 2006), as reprinted in Figure 2. In Figure 2, Our Inland North group was defined according to the region identified as Inland North in the Atlas of North American English (ANAE) (Labov et al., 2006, see ANAE page 148 map 11.15). The Inland North is the region around the US Great Lakes states and stretching east into New York state and also stretching downward along the “St. Louis Corridor” to St. Louis, following the ANAE analysis of this region as the Northern Cities Shift region. The Southern group was defined as speakers located in the traditional US South in the ANAE, not including Florida. Florida is exceptional since it has large amounts of immigration from northern US regions, and it has a different sociolinguistic history (controlled by Spain for a long period of time in the colonial era). Finally, our General North group was defined as all speakers not in the South, not in Florida, and not in the Inland North (and therefore, as roughly equivalent to Standard American English). This also includes Western varieties of American English. The reason for this analytical choice to define the General North broadly is that this broad region is known to contrast sharply both with the South and with the Inland North, as defined in the ANAE, in terms of two major vowels shifts considered here: The Southern Vowel Shift and the Northern Cities Shift. That is, in prior work (ANAE, Labov et al., 2006) the SVS vowel features were found in the South as defined here, and speakers in this region contrasted with speakers elsewhere in North America. Therefore, for SVS we compare speakers in the South group versus speakers in the General North group. As for NCS, the ANAE determined that the NCS vowel shift was found in the Inland North in contrast to the vowel system of the General North; the regional boundaries of the Inland North are defined in the ANAE in terms of this vowel shift that differs from the General North. Likewise, our NCS analysis compares Inland North speakers with General North speakers. In this way, we are able to test whether the NCS vowel contrast that the ANAE reported in terms of Inland North versus General North, which was based on manual vowel extractions, is also present in the IDEA data set using the automated methods of our present paper.

Once the recordings are transcribed, we calculated the Character Error Rate between (i) the ground truth transcription and the DeepSpeech automatic transcription and (ii) the ground truth and the CMU Sphinx transcription (see *Character Error Rate* below). We then extracted the formants of the vowels in each transcription system (ground truth, Sphinx, and DeepSpeech) with the DARLA semi-automated system, which uses the Montreal Forced Aligner and FAVE-Extract to calculate the formant information. This workflow is shown in Figure 1 above. We then constructed vocalic triangles, diagrams of the position of different vowels along F1 and F2. We then compared the positions of different vowels according to F1 and F2 and measured the degree of overlap between the ground truth vowels and both the Sphinx and the DeepSpeech transcribed vowels. Finally, we used this information to observe two well-known phenomena in American English: Southern Vowel Shift and the Northern Cities Vowel Shift.

Following standard methods in American English sociophonetics, we removed tokens of vowels in unstressed and reduced syllables since such tokens do not accurately represent the vowels being studied here (Thomas, 2011). Likewise, we removed tokens of vowels in function words (e.g., common grammatical words like “the,” “and,” etc.), and also any tokens with large, unreliable formant bandwidths (greater than 300 Hz). This filtering of high-bandwidth tokens is a standard way of ensuring that the tokens used in the study are based on reliable Linear Predictive Coding, since their LPC formant estimations are likely to be less reliable at a high-bandwidth (Hofmann, 2014:110, 162, 196; Ladefoged, 2003:117; Thomas, 2011:47). To reduce the effects on varying phonetic environments, we also removed tokens where vowels are in pre-liquid position, following standard practice for such shifts (Fridland and Bartlett, 2006; Nesbitt, 2018). Finally, since physiology and other factors can affect vocal tract length and vowel formants, we normalized the vowel formant measurements using the Lobanov method (Lobanov, 1971; Kendall and Thomas, 2010). The Lobanov normalization method has been one of the more commonly used approaches in sociophonetics spanning a large amount of time up to the present (e.g., Thomas, 2011; Fridland et al., 2014; Grama and Kennedy, 2019; Fridland and Kendall, 2019; D'Onofrio and Van Hofwegen, 2020; Nesbitt, 2021). We recognize that Barreda’s perceptual analyses (Barreda, 2020, Barreda, 2021) suggest a log-based method rather than Lobanov, and future work may take that approach. However, the prior DARLA testing (Reddy and Stanford, 2015a; Reddy and Stanford, 2015b) that we are comparing in the present study used the Lobanov method, and we prefer a direct comparison between the results here and previous ones. We also note that the Lobanov normalization is included in the FAVE output spreadsheets, and so computational sociolinguistics readers will be familiar with this output. We also note that there are a large number of different vowel normalization practices and debates in sociolinguistics (see Thomas and Kendall, 2007 online NORM site for detailed discussion of five such methods). We decided to use one of the more commonly accepted methods at the present time, the Lobanov method, recognizing that every method has its own strengths and weaknesses.

## Results

This section compares the two transcription methods we used (Sphinx and DeepSpeech) in the following ways: (i) How well their transcriptions overlap with manual transcriptions (*Character Error Rate*), and (ii) how effective they are in detecting sociolinguistic phenomena such as the Southern Vowel Shift (*Sociophonetic Results From the Southern Vowel Shift*) and Inland North Cities Shift (*Northern Cities Shift Results*).

### Character Error Rate

In order to investigate the differences in error rate between the transcription methods, we used a linear mixed effects model with character error rate as the dependent variable. Character error rate (henceforth CER) is the edit distance between two strings. For example, if the ground truth had the transcription “BAT” and the ASR produced the transcription “CAT,” then the CER would be 0.33^4^. The CER was log-transformed to meet the assumptions of linear-mixed effects models. As for the independent variables, we used the type of transcription (DeepSpeech versus Sphinx, henceforth DS and SPH), the gender of the speakers, the geographic area (Inland North, Southern, and General North) the estimated year of birth (from 1915 to 2007), and the interaction between transcription type and gender. This interaction was included because research has shown that ASR systems perform systematically worse on female voices (Tatman and Kasten, 2017). All categorical variables were encoded using treatment coding; the reference level for each of them was the first one alphabetically (type of transcription: DeepSpeech, gender: female, area: Inland North). The numerical variable estimated year of birth was centered by calculating the z-score of the variable. Finally, the model included a random intercept for speakers.

The DeepSpeech ASR does produce a statistically significant improvement in transcription, and this improvement is greater for females than for males [transcription by gender interaction: β_Male:DS_ = −0.13 ± 0.03, t (347) = −4.0, *p* < 0.001]. As for the males, there is a reduction of 0.09 units in the character error rate (from CER_SPH/M_ = 0.46 ± 0.11 to CER_DS/M_ = 0.37 ± 0.17). On the other hand, the improvement is greater when transcribing speech from females. In this case, the reduction in character error rate is 0.13 units (from CER_SPH/F_ = 0.46 ± 0.13 to CER_DS/F_ = 0.33 ± 0.16). This result is particularly important given the known issues with transcription of female speech, and our results suggest that deep-learning algorithms may be closing the gender gap in ASR performance. The main effects are also significant, confirming the direction of the interaction: When all other factors are held constant, there is a main effect for gender [β_Male_ = 0.13 ± 0.04, t (492) = 3.3, *p* < 0.001]: Overall, the transcription for males has a higher error rate (CER = 0.41) than the transcription for females (CER = 0.39). Likewise, there is a main effect for transcription type [β_Sphinx_ = 0.40 ± 0.02, t (347) = 18.0, *p* < 0.00001]: On average, when all other factors are held constant, the transcription for Sphinx (CER = 0.46) had more errors than the DeepSpeech transcription (CER = 0.35). These main results should be interpreted in light of the interaction: These main effect results agree with the more general result that Sphinx has more errors than DeepSpeech for female speakers. As for the other variables in the model, there are no significant differences in CER by region of the recording: Inland North: 0.40, South: 0.41, General North: 0.39 (p_InlandNorth/GeneralNorth_ = 0.67, p_South/GeneralNorth_ = 0.15). There are no significant differences in CER by estimated year of birth either (*p* = 0.30). Finally, the random intercept for speakers explains a sizable portion of the remaining variance in the regression (var_speaker_ = 0.08, var_residual_ = 0.05). In summary, the DeepSpeech ASR does provide improvements in transcription, particularly for speech from female speakers.

In general, the reduction in CER is an indication that the DeepSpeech transcriptions are closer to the original. The examples below show the improvements in the transcriptions of a speaker from the Inland North region, specifically from Minnesota. While words like “mom” are mistranscribed by both systems (as man and men respectively), the DeepSpeech transcription produced the correct vowel in “me”/“he,” and correctly transcribed the words “that they do” and “so my.”

*GT*: So, my mom and me came down here for the orientation that they do.

*DS*: so my man and he came down here for the orientation that they do (CER = 0.15).

*Sphinx*: follow men and i mean came down here for the orientation of the u (CER = 0.34).

The examples below show transcriptions for speakers from the South, from Alabama and Louisiana respectively. In the Alabama example, the DeepSpeech transcription is completely correct, but the Sphinx transcription has a few problems, including missing the pronoun “I” and mistranscribing “born in Jackson County” as going to act in canton. The Louisiana example shows an example of audio that was grossly mistranscribed by both systems. Even though they both make mistakes, the DeepSpeech system is closer to the original. For example, the stressed vowels in the words “growing” and going are the same (OW), whereas the Sphinx transcription has ground for those segments, which has the vowel AW^5^.

Alabama:

*GT*: I was born in northeastern Alabama. I was born in Jackson County.

*DS*: I was born in north eastern alabama I was born in jackson county (CER = 0.08).

*Sphinx*: I was born in northeastern alabama was going to act in canton (CER = 0.28).

Louisiana:

*GT*: Growing up with my sister, I always felt like I got the short end of the stick.

*DS*: the going on with my sister always felt like so i got the short instink (CER = 0.33).

*Sphinx*: the ground and women does your always the white jacket distorted (CER = 0.73).

Given that there is a significant improvement in transcriptions, our next question is: Do these new transcriptions extend our capabilities to detect sociophonetic patterns in automatically transcribed data? We will test these by trying to observe the well-understood phenomena of two North American English vowel shifts: The Southern Vowel Shift and the Northern Cities Shift (Labov et al., 2006).

### Sociophonetic Results From the Southern Vowel Shift

In the first subsection we will present the improvements in the sociophonetic analysis of speakers of Southern English. Following this, we will compare the Southern speakers with those of the General North, and analyze how the DeepSpeech transcription performs in comparing these two.

#### Improvements in Sociophonetic Analysis

**FIGURE 3:**
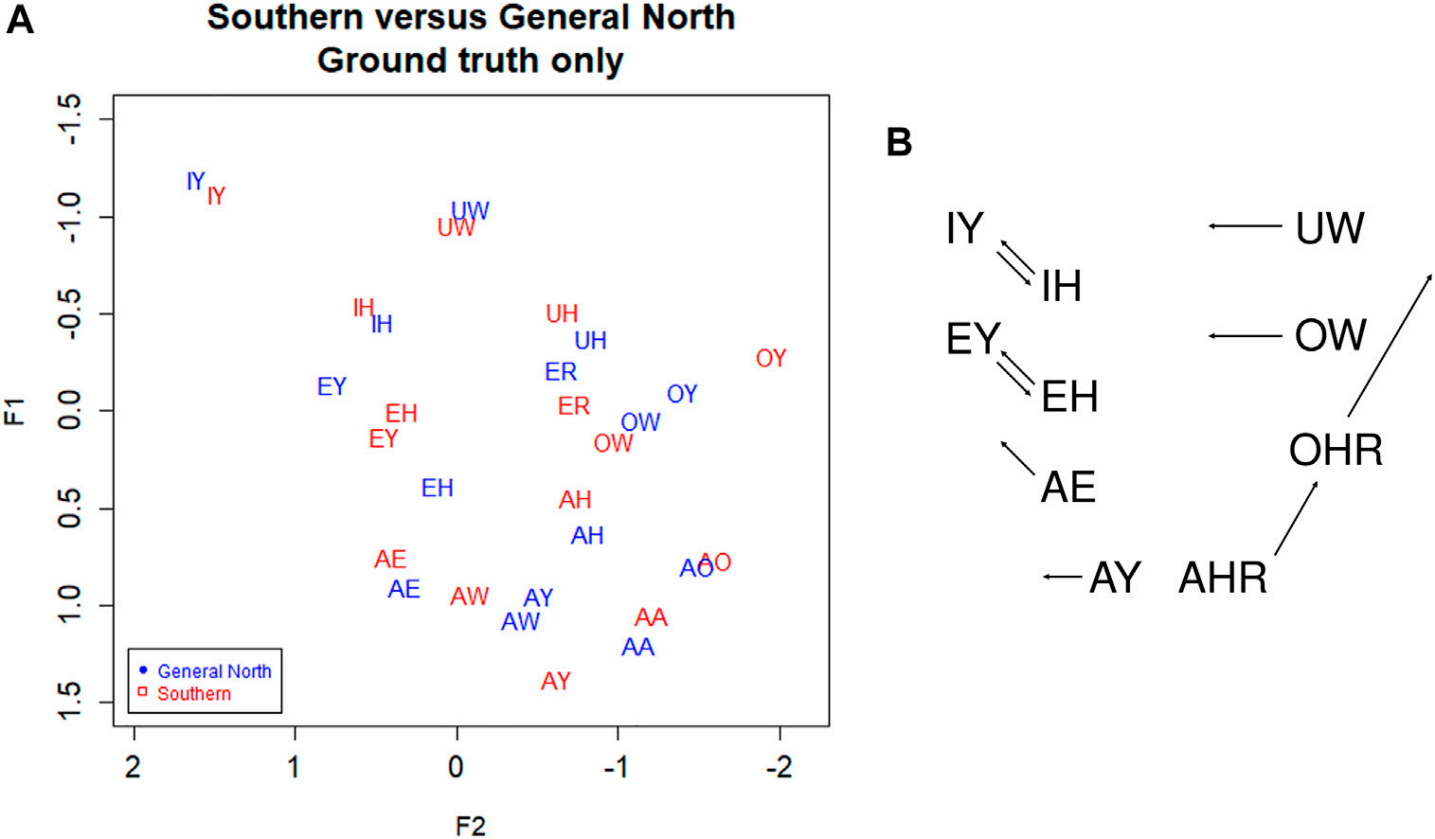
**(A)** Southern versus General North data from manual transcription: Red = South, Blue = North. Ground truth (manual transcription). Speaker vowel means. 330 speakers, 31,900 tokens. Plotted in Lobanov-normalized units. **(B)** Abstract schematic of the Southern Vowel Shift (adapted from Labov, 1996).

In Figure 3A, we plot the Southern speakers versus the General North speakers using the ground truth (GT) transcription. We can see that the Southern Vowel Shift (SVS) is evident in this manually transcribed version of the data. First, note the EY/EH tense/lax shift in the General South (red) speakers such that EH becomes higher than EY (compare to the SVS schematic in Figure 3B). We also see some graphical evidence for the IY/IH tense/lax shift, although we expect this to be a weaker shift. Next, we note a highly advanced AW vowel in the General South speakers, as well as evidence of AE raising and OW-fronting. We note UW-fronting as well, but this is shared by both the General North and Southern speakers, suggesting an overall pattern of UW-fronting. We do not examine AXR and other complex shifts involving liquids since such movements go beyond the scope of the present study; likewise, the Southern monophthongization of AY and raising of OY and so on are topics for another study since they would require analysis of the off-glide.

**FIGURE 4:**
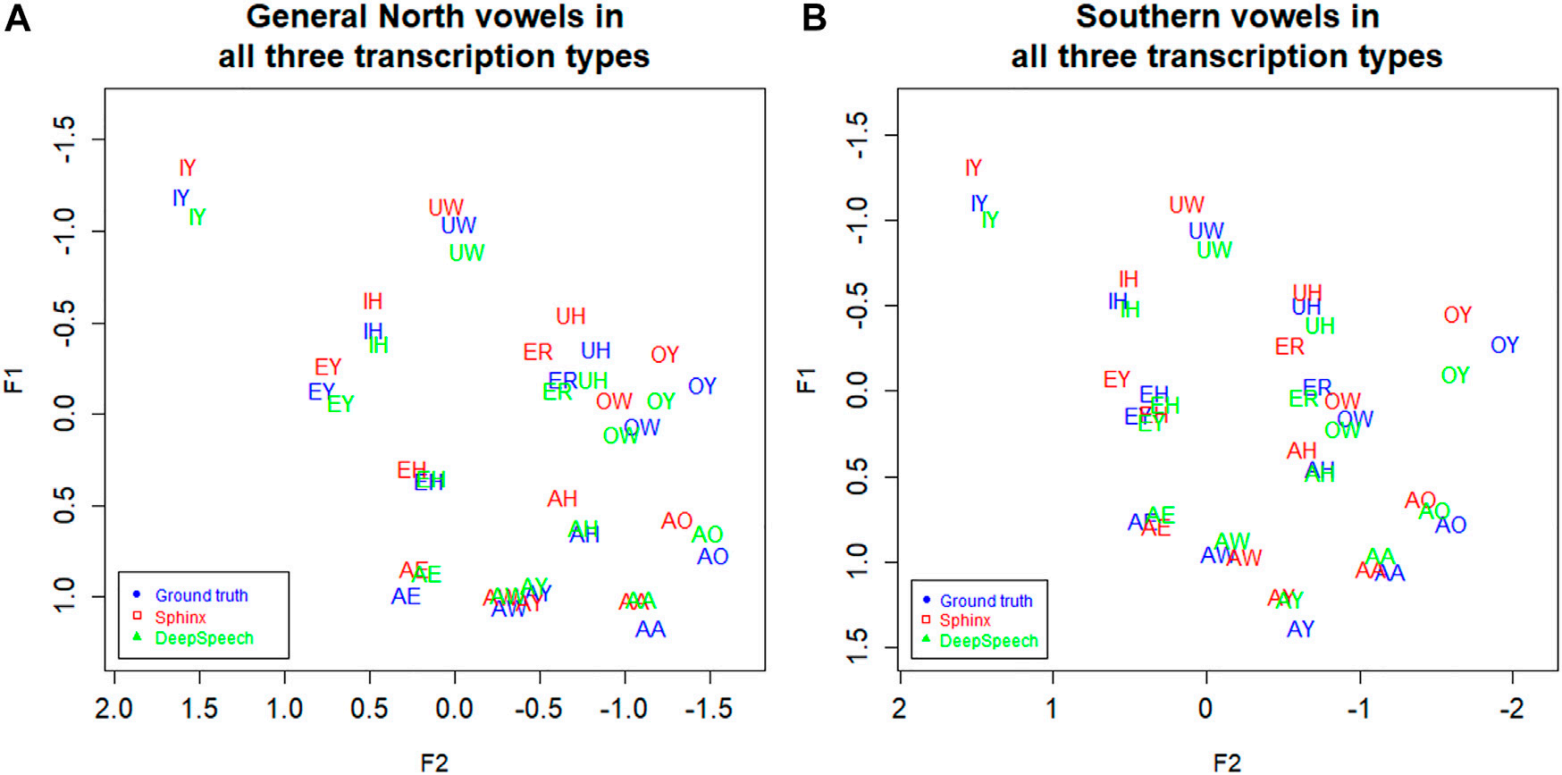
**(A)** General North and **(B)** Southern speaker’s vowels (mean position) in all three transcription types. Blue: Ground truth (manual) transcription, Red: CMU Sphinx transcription, Green: DeepSpeech transcription. 105 speakers and a total of 28,225 vowel tokens. Plotted in Lobanov-normalized units.

Now consider Figure 4B, which shows Southern speakers in all three of the transcription types. Figure 4A, which includes the three transcription types for General North, is included for comparison. All three of the transcription types show the SVS features noted above, but ground truth (GT) and DeepSpeech (DS) show the clearest differences between the two dialects. In particular, note the configuration of EY/EH for the three transcription types. Both ground Truth and DeepSpeech show the full rotation as EH and EY “switch places” in the vowel space, as we would expect from the schematic in Figure 3B: the EY vowel (the vowel in FACE or MADE) retracts and lowers, while EH (the vowel in DRESS or RED) fronts and raises. By contrast, DARLA’s current in-house Sphinx version only shows a general movement of EY/EH toward the SVS configuration but not the rotation. We expect that the Southerners’ EY/EH shift will be more advanced than their IY/IH shift because this is commonly the case for the Southern Vowel Shift (Kendall and Fridland, 2012), and this is what we find in the figure. Moreover, we find that the DeepSpeech version more accurately reflects the status of the tense/lax shift than the Sphinx version.

We now examine each of the vowels in the SVS in terms of F1 and F2, comparing across all three transcription types: DeepSpeech, DARLA’S CMU Sphinx, and the ground truth. Following Johnson (2015) and Stanley (2018), we compare the token distributions using Bhattacharyya’s Affinity (BA). This is calculated by describing each token by its two-dimensional coordinates (Lobanov-normalized F1 and F2), and then measuring the amount of overlap between the regions covered by both vowels. An affinity of 1.0 indicates a perfect overlap between the two distributions of vowel tokens, and an affinity of 0.0 indicates perfectly non-overlapping distributions. The formula and the concrete implementation used can be found in the kerneloverlap function in the R package adehabitatHR (R Core Team, 2021; Calenge, 2006). We use Bhattacharyya’s Affinity rather than Pillai approaches since Johnson (2015) argues that BA improves upon Pillai by more accurately quantifying overlap for the purposes of vowel distributions (for example, by better handling unequal distributions or distributions with an unequal number of tokens). In addition to this, Bhattacharyya’s Affinity has been used to study vowel contrasts in New Zealand English (Warren, 2018) and in back vowels in Kansas (Strelluf 2016).

For each of the Southern Vowel Shift vowels, we compute the Bhattacharyya’s Affinity for each speaker’s distribution in terms of Sphinx versus ground truth, and then in terms of DeepSpeech versus ground truth. We then use a repeated-measures ANOVA to determine the relationship between Bhattacharyya’s Affinity, type of transcription and the vowels in the transcripts. The affinity was used as the dependent variable, transformed with a reflected square root transformation to comply with normality assumptions. The vowels and the types of transcriptions were used as within-subjects independent variables.

**FIGURE 5:**
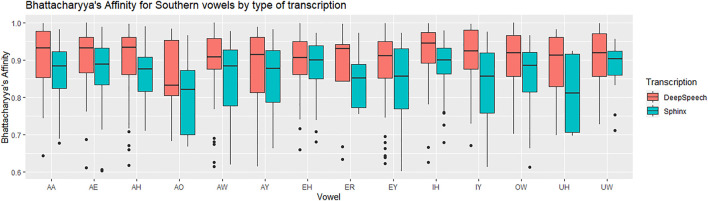
Southern data: vowel medians for the Bhattacharyya’s affinity by type of transcription. Red: DeepSpeech versus ground truth, Green = CMU Sphinx versus ground truth. 105 speakers and a total of 28,225 vowel tokens.

There was a significant difference between the Sphinx transcription and the DeepSpeech transcription [F (1) = 25.8, *p* < 0.00005, η^2^ = 0.053]. As can be seen in Figure 5, the vowels transcribed with DeepSpeech have a higher BA with the ground truth vowels. The median affinity for CMU Sphinx is 0.88, while the median affinity for DeepSpeech is 0.92. There was also a significant difference between vowels [F (13) = 1.2, *p* < 0.00005, η^2^ = 0.034]: Some vowels have higher overall BAs (e.g., EH: 0.901, EY: 0.889, IH: 0.916, IY: 0.894), while others have significantly lower affinities (e.g., AO: 0.817). Table 1 shows the vowels involved in the SVS. The interaction between vowels and type of transcription was not statistically significant (*p* = 0.26), meaning that no vowels were observed to have a marked improvement over others. In general, for all of the vowels involved in the SVS there is a gain in BA when transcribed automatically using DeepSpeech.

**TABLE 1:** Bhattacharyya’s Affinity for DeepSpeech versus ground truth and Sphinx versus ground truth in Southern speakers. BA score 1.0 = perfectly overlapping distributions, 0.0 = completely non-overlapping.

SVS vowel	Median BA for DS vs. GT	Median BA for SPH vs. GT	Δ(BA)
AW	0.907	0.883	0.024
EH	0.905	0.899	0.006
EY	0.910	0.855	0.055
IH	0.943	0.899	0.043
IY	0.923	0.850	0.074
OW	0.916	0.880	0.036
UW	0.918	0.903	0.015

#### Variationist Analysis of SVS Movements

**FIGURE 6:**
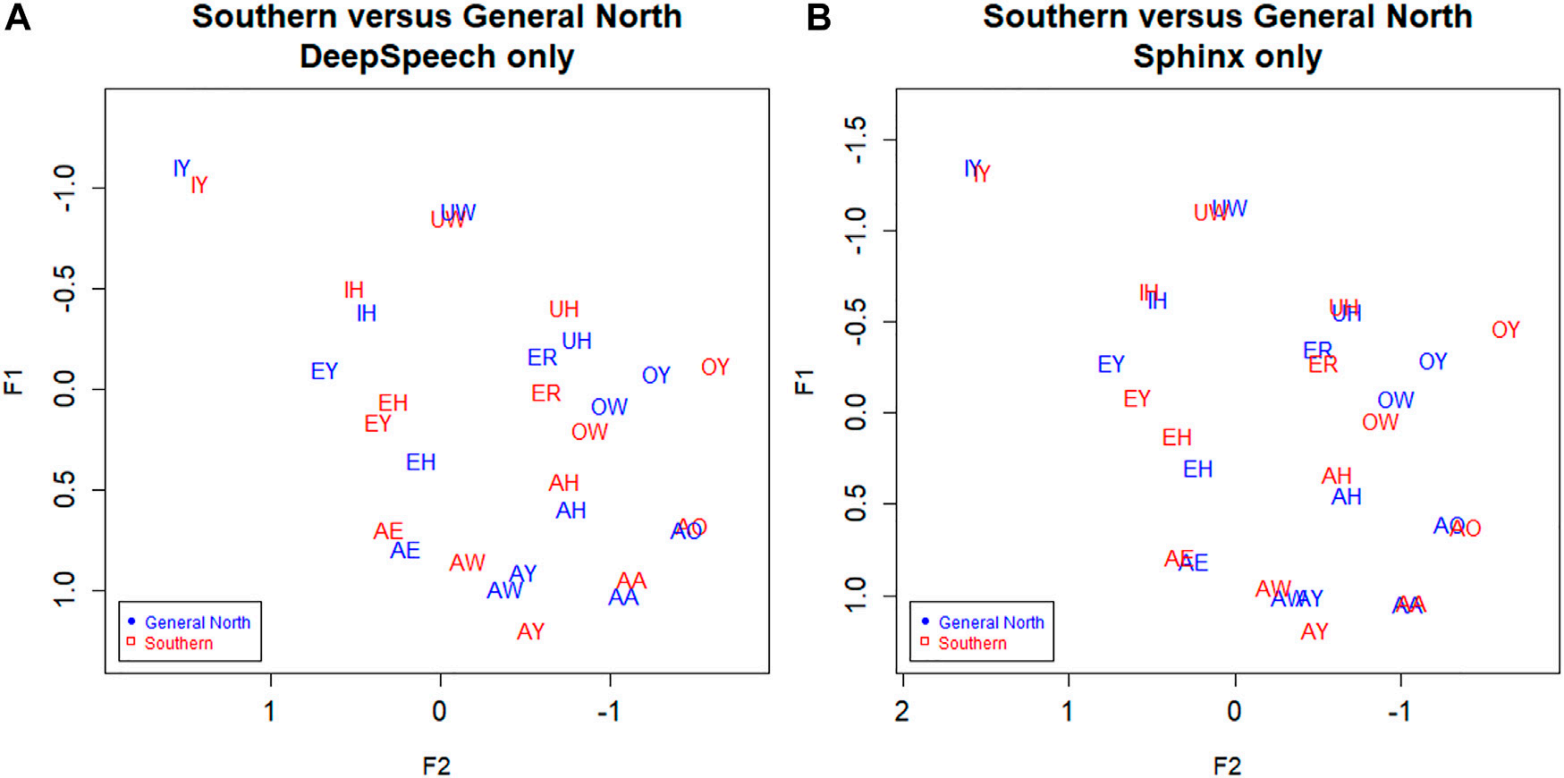
Southern versus General North. Red: General North, Red: Southern. **(A)** DeepSpeech ASR, speaker vowel means. 330 speakers, 24,295 tokens. **(B**) CMU Sphinx ASR, speaker vowel means. 330 speakers, 27,637 tokens. Plotted in Lobanov-normalized units.

In the previous section we presented evidence that the DeepSpeech-based system is more effective than Sphinx at measuring the vowels of Southern speakers. Based on this, we can assume that the DeepSpeech system will also help in observing the vowel differences between Southern speakers and speakers of the General North variants. Figure 6A below shows the vowels from these two dialects, as extracted from the DeepSpeech data. Compare this to Figure 6B, the vowels as extracted by the Sphinx ASR system. The DeepSpeech system shows a clearer impressionistic separation between the two dialects. For example, the vowel IY shows a much clearer separation in the DeepSpeech data (Figure 6A), compared to the partial overlap in the Sphinx data (Figure 6B).

The next step is to conduct a variationist analysis of all of the major movements of the Southern Vowel Shift. Using linear mixed effects modeling with the lme4 package (Bates et al., 2015) from (R Core Team, 2021), we built models with the independent variables of Region (General North versus South), Year of Birth,^6^ Gender, and Following Environment (nasal, voiceless obstruent, voiced obstruent). Year of birth is a numerical variable, so it was centered using z-scoring; the categorical variables were encoded using treatment coding. We also included the variable Transcription type (DeepSpeech versus ground truth), so that we can examine how well DeepSpeech holds up in comparison to the ground truth. The interaction between transcription type and region was also included, to determine whether the DeepSpeech automated transcription shows the North/South differences in a way that is, similar to the ground truth data (for example, by seeing of the degree of separation between Northern and Southern IY in the ground truth is also present in the DeepSpeech data). Finally, the election of the random effects for each model proceeded *via* backward selection from a maximal model, which included all variables (as well as the Region:Transcription interaction) for both speaker and word effects (Barr et al., 2013; Bates et al., 2015). We used the step instruction in (R Core Team, 2021), which gave us an optimal random effect structure for each of the vowels. The resulting models are shown in Supplementary Appendix S1.

The dependent variable for these models will vary according to the relevant variable for the motion of each vowel. For example, some vowels, like AE, show raising, which is quantified using the standard sociophonetic formula from Labov et al. (2013) 40 which describes such diagonal movement along the front of the vowel space by the relationship: F2 - (2 × F1). Other vowels, such as AW, will use Lobanov-normalized F2 as the dependent variable, to show their movement front or back. All of the dependent variables were transformed (arcsin of the square root) to improve normality and meet the assumptions of LMERs. In summary, the linear mixed-effects modeling will provide 1) a basic description of the Southern Vowel Shift in the data set and 2) a quantified way of determining how close the DeepSpeech transcription gets to the ground truth version. In other words, using publicly available speech recognition methods (like Mozilla DeepSpeech), how close have we come to being able to produce a reliable “hands-free” analysis of a vowel shift from fieldwork recordings of conversations?

**FIGURE 7:**
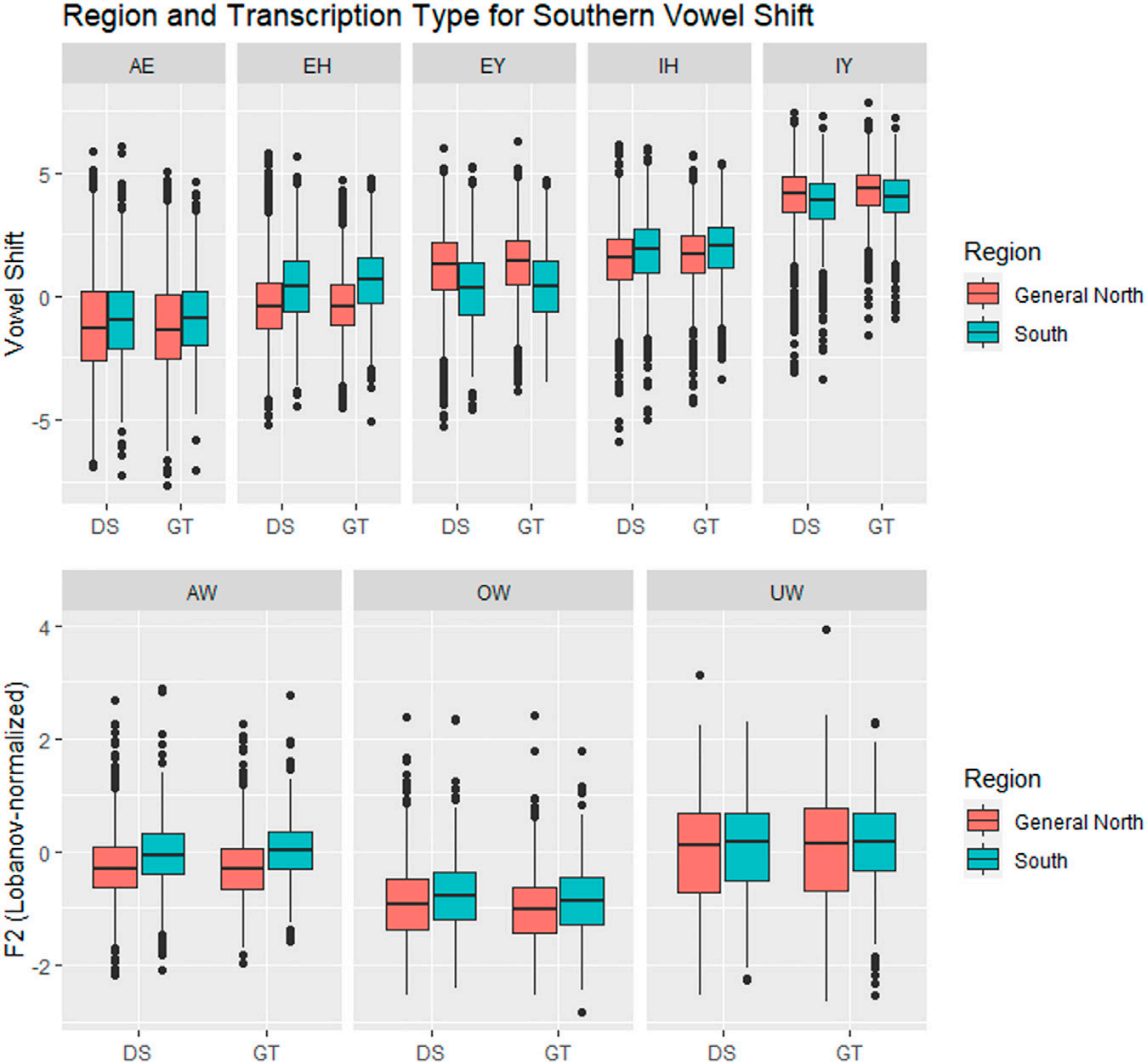
Vowels in the Southern Vowel Shift, by Region (General North versus Southern) and transcription type. In six of the vowels (AW, EH, EY, IH, OW, UW) there is a significant separation between General North and Southern vowels, and this is tracked by both transcription systems. DS = DeepSpeech, GT = Ground truth.

Table 2 shows the results from the models. First, we will examine the results related to Region and Transcription type. The most relevant result is that, for seven out of eight vowels involved in the shift, the behavior of the DeepSpeech data is similar to that of the ground truth data. Figure 7 shows the vowel shift and the F2 for the vowels involved in the Southern Vowel Shift, separated by transcription type. There are five of the vowels, AW, EY, EH, IH, OW, where there are clear differences between the North and South tokens, and these are visible in both the DeepSpeech and the ground truth data. For the vowel AW, for example, the North/South difference for the ground truth is ΔRegion_GT_ = 0.31, whereas the North/South difference for DeepSpeech transcriptions is ΔRegion_DS_ = 0.22. The model as a whole shows differences between North and South [β_South_ = 0.03 ± 0.01, t (179) = 2.9, *p* < 0.005]. The model also shows a significant interaction between Region and Transcription [β_GT:South_ = 0.02 ± 0.007, t (2567) = 2.2, *p* < 0.05], which means that the ΔRegion_GT_ = 0.31 is significantly smaller than the ΔRegion_DS_ = 0.22. A estimated marginal means (EMM), Tukey-corrected post-hoc analysis was carried out to determine if each of those deltas was actually significantly different from zero (i.e., is there a significant difference between North/South if we looked just at the DeepSpeech data, or if we looked just at the ground truth data?). This was calculated using the emmeans package in R (Russell, 2021 ). The post-hoc results in Table 2 confirm that, in the case of AW, both the ground truth (z = −4.8, *p* < 0.0001) and DeepSpeech transcriptions (z = −2.9, *p* < 0.05) show significant differences between North and South. Taken together, these results indicate that, even if DeepSpeech sees less of a difference between the Northern and Southern tokens of AW, it still sees a significant difference between them, and therefore, the ground truth and DeepSpeech data are describing this sociolinguistic variation is roughly similar ways.

**TABLE 2:** LMER models for comparison between Southern Vowel Shift (SVS) and General North vowels. The dependent variable is F2 for the vowels mouth (AW), goat (OW), goose (UW), and F2-2xF1 for the vowels trap (AE), face (EY), dress (EH), fleece (IY), and kit (IH). *R* ^2^ shows marginal and conditional coefficient. Deltas show the difference between the mean position of the Northern vowel and the mean position of the Southern vowel for each transcription type: AE shows divergence in GT/DS results; for the other vowels, either both models detect a North/South difference or they do not.

A. Results for region and transcription type; *R* ^2^ for entire model						
**Vowels**	**Region by Transcription**	**Region**	**Transcription**	**Post-hoc ΔRegion_GT_**	**Post-hoc ΔRegion_DS_**	***R* ^2^**
AE (*n* = 5198)	*p* = 0.08	*p* = 0.09	β_GT_ = −0.001 ± 0.0004 t (265) = −2.6, *p* < 0.01	Δ = 0.40 z = −3.0, *p* < 0.05	Δ = 0.28 *p* = 0.33	0.21
						0.69
AW (*n* = 3116)	β_GT:South_ = 0.02 ± 0.01 t (2567) = 2.2, *p* < 0.05	β_South_ = 0.03 ± 0.01 t (179) = 2.9, *p* < 0.005	β_GT_ = 0.03 ± 0.008 t (52) = 3.4, *p* < 0.005	Δ = 0.31 z = −4.8, *p* < 0.0001	Δ = 0.22 z = −2.9, *p* < 0.05	0.10
						0.56
EY (*n* = 5730)	*p* = 0.41	β_South_ = −0.07 ± 0.01 t (179) = −7.8, *p* < 0.00001	β_GT_ = 0.02 ± 0.004 t (201) = 4.7, *p* < 0.00001	Δ = 0.86 z = 7.9, *p* < 0.0001	Δ = 0.84 z = 7.8, *p* < 0.0001	0.08
						0.59
EH (*n* = 5930)	β_GT:South_ = 0.02 ± 0.01 t (144) = 2.0, *p* < 0.05	β_South_ = 0.06 ± 0.01 t (121) = −1.3, *p* < 0.00001	*p* = 0.20	Δ = 0.98 z = −9.1, *p* < 0.0001	Δ = 0.75 z = −6.2, *p* < 0.0001	0.10
						0.53
IY (*n* = 4442)	*p* = 0.86	*p* = 0.13	β_GT_ = 0.03 ± 0.005 t (134) = 7.2, *p* < 0.00001	Δ = 0.29 *p* = 0.15	Δ = 0.26 *p* = 0.43	0.08
						0.55
IH (*n* = 5961)	*p* = 0.58	β_South_ = 0.03 ± 0.008 t (282) = 4.0, *p* < 0.00001	β_GT_ = 0.02 ± 0.003 t (333) = 5.2, *p* < 0.00001	Δ = 0.28 z = −4.2, *p* < 0.0005	Δ = 0.30 z = −4.0, *p* < 0.0005	0.02
						0.62
OW (*n* = 4611)	*p* = 0.25	β_South_ = 0.03 ± 0.009 t (250) = 3.2, *p* < 0.005	β_GT_ = −0.03 ± 0.004 t (69) = −7.2, *p* < 0.00001	Δ = 0.16 z = −4.5, *p* < 0.0001	Δ = 0.12 z = −3.2, *p* < 0.01	0.03
						0.50
UW (*n* = 2786)	*p* = 0.70	β_South_ = 0.02 ± 0.01 t (255) = 2.1, *p* < 0.05	*p* = 0.25	Δ = 0.08 *p* = 0.07	Δ = 0.06 *p* = 0.17	0.01
						0.56
**B. Results for other social and linguistic variables in the model**						
**Vowels**	**Year of birth**	**Gender**	**Following environment (Nasal versus voiced obstruent)**		**Following environment (Nasal versus voiceless obstruent)**	
AE (*n* = 5198)	β = −0.001 ± 0.0003, t (195) = −2.3, *p* < 0.05	*p* = 0.54	β_Nas/+VoicedObs_ = −0.12 ± 0.01, t (280) = −9.7, *p* < 0.00001		β_Nas/-VoicedObs_ = −0.15 ± 0.001, t (525) = −15.7, *p* < 0.00001	
AW (*n* = 3116)	β = −0.02 ± 0.004, t (246) = −4.6, *p* < 0.00001	*p* = 0.87	β_Nas/+VoicedObs_ = −0.06 ± 0.01, t (80) = −4.7, *p* < 0.0001		β_Nas/-VoicedObs_ = −0.06 ± 0.01, t (108) = −4.5, *p* < 0.0001	
EY (*n* = 5730)	β = 0.02 ± 0.004, t (171) = 5.2, *p* < 0.00001	*p* = 0.65	*p* = 0.34		β_Nas/-VoicedObs_ = 0.03 ± 0.01, t (196) = 2.4, *p* < 0.05	
EH (*n* = 5930)	β = −0.01 ± 0.003, t (283) = −3.2, *p* < 0.005	β_male_ = 0.02 ± 0.007, t (283) = −3.2, *p* < 0.0005	β_Nas/+VoicedObs_ = −0.03 ± 0.01, t (434) = −3.2, *p* < 0.005		β_Nas/-VoicedObs_ = −0.06 ± 0.01, t (495) = −7.2, *p* < 0.00001	
IY (*n* = 4442)	β = 0.01 ± 0.003, t (257) = 4.3, *p* < 0.00001	*p* = 0.15	β_Nas/+VoicedObs_ = 0.06 ± 0.01, t (115) = 5.4, *p* < 0.00001		β_Nas/-VoicedObs_ = 0.08 ± 0.01, t (132) = 6.9, *p* < 0.00001	
IH (*n* = 5961)	β = 0.005 ± 0.002, t (286) = 2.2, *p* < 0.05	*p* = 0.32	*p* = 0.17		β_Nas/-VoicedObs_ = 0.02 ± 0.01, t (424) = 2.2, *p* < 0.05	
OW (*n* = 4611)	β = 0.009 ± 0.003, t (297) = 2.7, *p* < 0.01	*p* = 0.99	*p* = 0.30		*p* = 0.13	
UW (*n* = 2786)	β = 0.01 ± 0.003, t (265) = 3.1, *p* < 0.005	*p* = 0.79	*p* = 0.10		*p* = 0.76	

The same general patterns observed for AW are also present in the vowels EY, EH, IH, and OW. In all of them there is a significant difference between Region, which means that the North/South differences were visible in the data. The post-hoc analysis also indicates that the North/South difference can be found in both the ground truth and the DeepSpeech data, and only in the EH is there an interaction between Region and Transcription: The North/South difference in ground truth (ΔRegion_GT_ = 0.86) is significantly larger than the difference in DeepSpeech [ΔRegion_DS_ = 0.75, β_GT:South_ = 0.02 ± 0.01, t (144) = 2.0, *p* < 0.05]. In the other three vowels (EY, IH, and OW), both the ground truth and the DeepSpeech data have a similar magnitude for the North/South difference.

There are two vowels, IY and UW, for which neither the ground truth nor the DeepSpeech data could find a significant difference between North and South. (UW has a main effect for Region, but this is an additive effect when both types of transcriptions are put together; once they are separated by the post-hoc test, the significance disappears, with *p* = 0.07 for ΔRegion_GT_ and *p* = 0.17 for ΔRegion_DS_). This means, in essence, that both ground truth and DeepSpeech data fail to show Northern/Southern differences in similar ways.

The vowel AE deserves special mention because it is the one vowel where ground truth data shows a North/South difference, but DeepSpeech does not. There is a significant difference between transcriptions [β_GT_ = −0.001 ± 0.0004, t (265) = −2.6, *p* < 0.01], which is confirmed when the post-hoc results are computed: The ground truth shows a significant difference between North/South AE (ΔRegion_GT_ = 0.40, z = −3.0, *p* < 0.05). However, the DeepSpeech data does not show a significant difference in the tokens of AE in the two regions (ΔRegion_DS_ = 0.28, *p* = 0.33). This means that there was one vowel for which DeepSpeech and ground truth disagree. On the other hand, for the other seven, the results from the two transcription types are similar: Either both systems detect a difference, or neither of them does.

The bottom part of Table 2 also shows results which correspond to patterns that are well established in the sociophonetic literature on the Southern Vowel Shift (Labov et al., 2006). All of the vowels show significant effects for year of birth: For five of the vowels (EY, IY, IH, OW, and UW) younger speakers show more shift, whereas in three of them (AE, AW, and EH), older speakers show more shift. In six of the vowels there are significant differences in shift influenced by the phonological environment of the vowel (e.g., vowel followed by a nasal, a voiced obstruent like/g/, or a voiceless obstruent like/k/). Finally, only one of the vowels (EH) showed a significant difference by gender: Male speakers had a more negative shift (−0.17), whereas female speakers had a more positive shift (0.07).

Table 2 above shows the coefficient of determination (*R* ^2^) for the models used. The column shows the marginal correlation coefficient (from the fixed factors) as well as the conditional correlation coefficient (from both the fixed and random factors). The differences between the two show how much of the variation can be explained through random variation due to individual speakers and words: The marginal correlation, which is the correlation from the independent variables, ranges from *R* ^2^ = 0.01 to *R* ^2^ = 0.21. On the other hand the conditional correlation, which incorporates the random factor structure, can reach much higher correlation values, up to *R* ^2^ = 0.69 for the vowel AE, for example. This pattern is to be expected, given that the model doesn’t include numerous other factors that could explain variation across speakers (e.g., ethnicity) and variation across words (e.g., lexical frequency). The Supplementary Appendix includes the full results for the random variable structure of each model.

In summary, the data from the DeepSpeech automated transcription appears to be adequate in the detection of the SVS vowel patterns. While it is not perfect, it produces similar results to those from manually transcribed data. In the next section we will present evidence of the usability of the DeepSpeech data by focusing on a second sociolinguistic phenomenon, one that has not been extensively studied using automated methods: the vowel shift present in the Northern Cities of the United States.

### Northern Cities Shift Results

**FIGURE 8:**
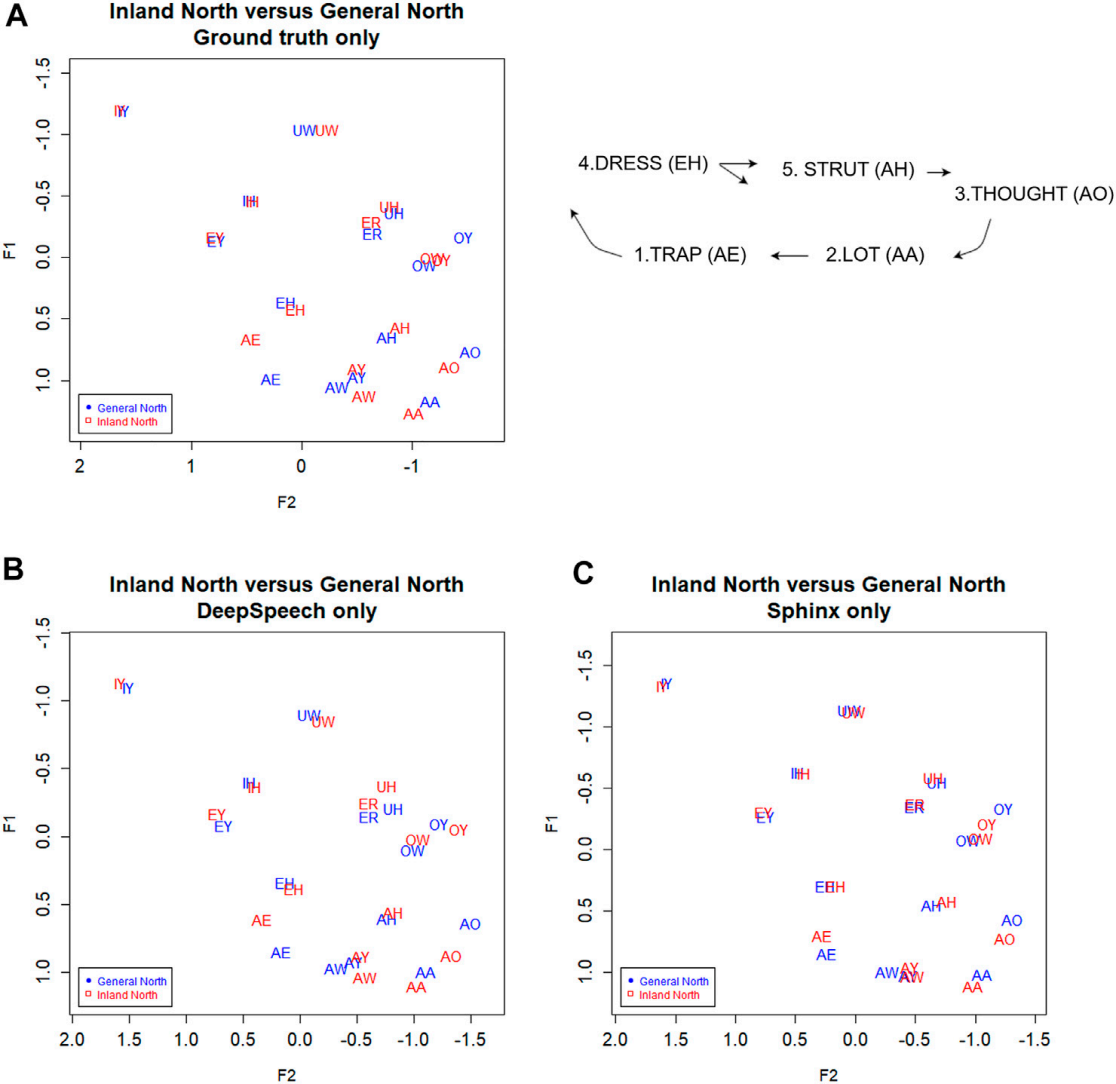
Inland North (red) and General Northern speakers (blue) in the **(A)** ground truth transcription, and transcribed automatically by **(C)** DeepSpeech and **(D)** Sphinx. Speaker vowel means for 225 speakers, 21,200 vowel tokens. Plotted in Lobanov-normalized units. **(B)** Schematic of the primary movements of the Northern Cities Vowel Chain Shift (adapted from Nesbitt, 2018).

Figure 8A shows the vowel means of IDEA speakers from Inland North (blue) and the General North regions (red). The General North speakers were defined as all speakers not from Inland North and not from the US Southern regions. In this plot of the results from the ground truth transcription, we see graphical evidence of the five classic NCS vowel movements (Labov et al., 2006; Nesbitt, 2018). First, note that AE has raised for Inland North speakers, representing the classic Stage 1 of the NCS. Second, the Inland North speakers appear to have fronted the AA vowel, which is NCS Stage 2. Then, in the classic chain shift model, the AO vowel has moved toward the original location of the AA vowel, which is Stage 3. For Stage 4, we see that the EH vowel appears to have moved down and back, and for Stage 5, we see that the AH vowel appears to have moved back.

Now consider Figure 8C, where we plot the Inland North versus General North again but this time we show the results for DeepSpeech and for the Sphinx ASR transcriptions. Overall, we observe the same NCS shifts in these automated transcription types, with similar directions and magnitudes (e.g., raising of AE). This indicates that the automated methods may be able to uncover the presence of the NCS in these recordings. On the other hand, there are differences between DeepSpeech and Sphinx: Vowels like IY and UW are almost completely overlapping in Sphinx (Figure 8D), whereas they show some separation in the DeepSpeech data. In order to test the differences between these, we will first compare Bhattacharyya’s Affinity between ground truth/DeepSpeech and ground truth/Sphinx to confirm the improvements from the DeepSpeech transcription. We will then use a linear mixed effects model to confirm that the DeepSpeech data correctly portrays the motions involved in the NCS.

#### Improvements in Sociophonetic Analysis

**FIGURE 9:**
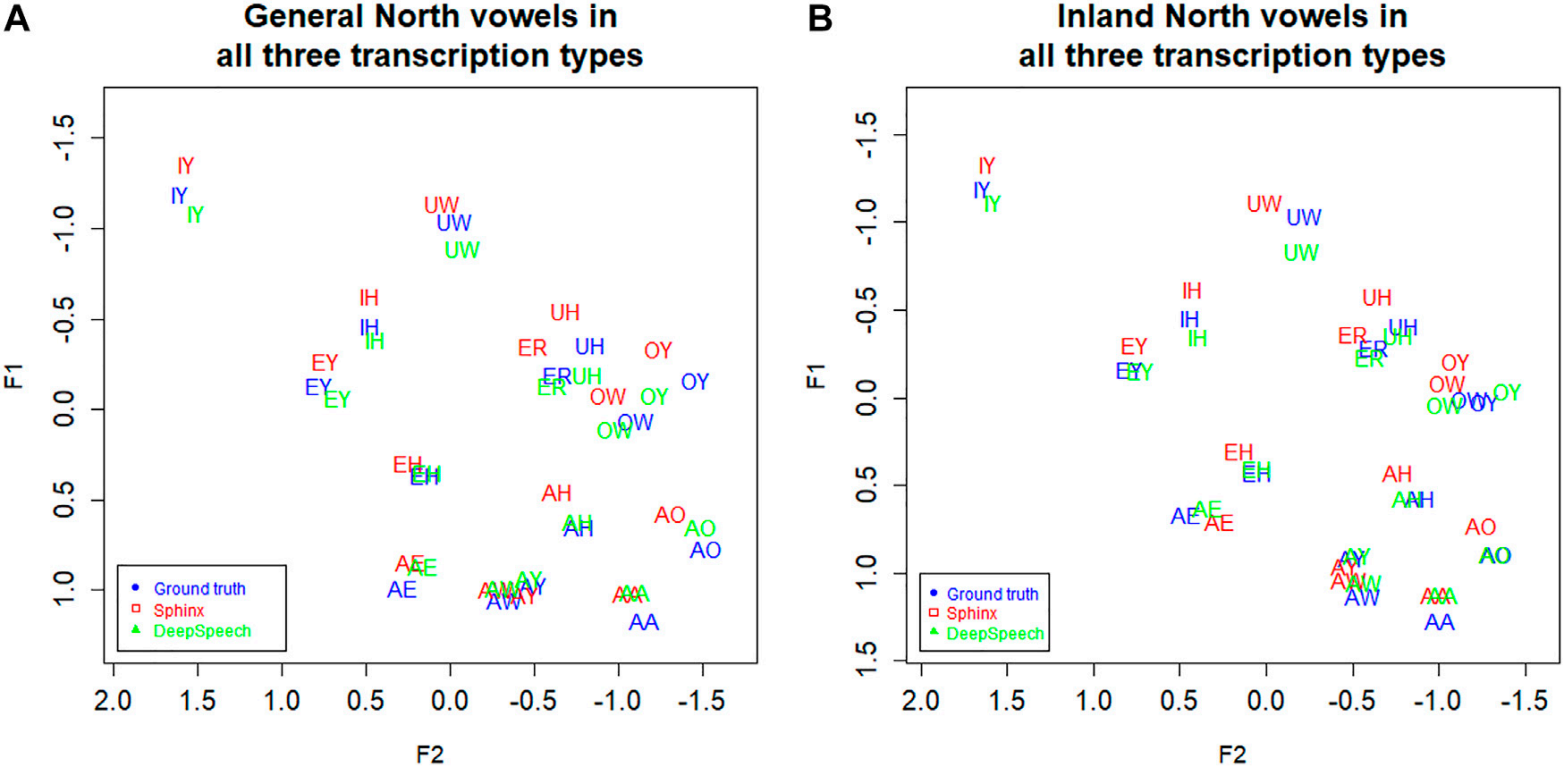
**(A)** General North and **(B)** Inland North: speaker vowel means in all three transcription types. Blue: Ground truth (manual) transcription, Red: CMU Sphinx transcription Green: DeepSpeech transcription. 58 speakers and a total of 14,414 vowel tokens. Plotted in Lobanov-normalized units.

We noted in Figure 8C that the DeepSpeech system shows graphical evidence of all the same NCS movements as we found above in the ground truth transcription: Raised AE, fronted AA, lowering of AO and EH, and backing of AH. To further the comparison of the transcription methods, all three transcription methods are plotted together in Figure 9B below. (Figure 4A, the transcription of the General North vowels, is repeated below as 9a for comparison).

**FIGURE 10:**
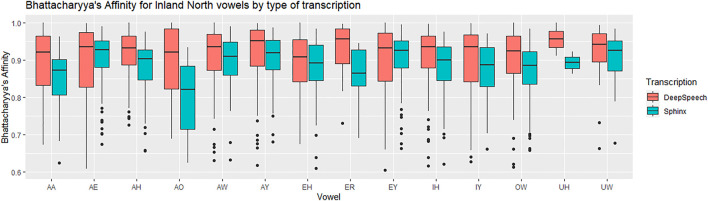
Inland North: vowel medians for the Bhattacharyya’s affinity by type of transcription. Red: DeepSpeech versus ground truth, Green = CMU Sphinx versus ground truth. 58 speakers and a total of 14,414 vowel tokens.

Next, we examine the difference in the NCS vowel transcription statistically. As with the SVS above, we calculated Bhattacharyya’s Affinity on Sphinx vs. ground truth and DeepSpeech vs ground truth, and then performed a repeated-measures ANOVA test to determine the effect of transcription and vowels on the BA. We used the same ANOVA structure, with the reflected square root corrected BA as the dependent variable, and vowels and type of transcription as independent, within-subjects variables. There was a significant interaction between vowels and transcription [F (13) = 2.9, *p* < 0.0005, η^2^ = 0.01] and there is a significant main effect for vowels [F (13) = 9.5, *p* < 0.0005, η^2^ = 0.036]. This means that there are BA differences between the vowels, and that some vowels benefit more from the DeepSpeech transcription than others. Figure 10 shows that vowels like AO have a high gain in BA in the DeepSpeech transcription (BA = 0.92 for DeepSpeech but BA = 0.80 for Sphinx). On the other hand, vowels like EY show practically no difference in their Bhattacharyya’s affinity, regardless of the transcription mechanism (BA = 0.93 for both DeepSpeech and Sphinx).

There is also a main effect for type of transcription: vowels transcribed with DeepSpeech show higher BAs with the ground truth [F (1) = 29.0, *p* < 0.00005, η^2^ = 0.025]. In general, the median affinity for DeepSpeech vowels is 0.93, while the median for Sphinx vowels is 0.90. Also, as can be seen in Table 3, the vowels involved in the Northern Cities Shift show improvement when transcripted using DeepSpeech. Vowels like EH and AH show only modest differences, whereas the vowel AO shows marked improvement.

**TABLE 3:** Bhattacharyya’s Affinity for DeepSpeech versus ground truth and Sphinx versus ground truth in Inland Northern speakers. BA score 1.0 = perfectly overlapping distributions, 0.0 = completely non-overlapping.

NCS vowel	Median BA for DS vs. GT	Median BA for SPH vs. GT	Δ(BA)
AA	0.916	0.869	0.047
AE	0.934	0.926	0.007
AH	0.931	0.903	0.028
AO	0.920	0.802	0.118
EH	0.907	0.891	0.016

#### Variationist Analysis of NCS Movements

Given the evidence that DeepSpeech is significantly better than CMU Sphinx at transcribing vowels from the Northern Cities Shift, we conducted a linear mixed-effects model analysis of the vowels from the Inland North, comparing DeepSpeech against the baseline ground truth. We use a similar linear mixed-effects structure as in the Southern Vowel Shift above: The fixed variables are Region, Transcription type, Year of birth, Gender, Following environment and the interaction of Region and Transcription type. The random effect structure was also chosen through backward selection using the step procedure; the resulting models are shown in the Supplementary Appendix.

Following the ANAE (Labov et al., 2006) and Labov (2013:40), we quantify the Northern Cities Shift movements of AA, AH and AO in terms of F2 to characterize fronting. For the diagonal movement of AE along the front of the vowel trapezoid, we follow the method in Labov et al. (2013) 40 of using the equation F2 - (2 × F1) to create a single numerical value representing the raising and fronting. We use the same equation to account for movements of the other front vowel, EH, since the NCS movement of EH may be either backing or lowering or both, as seen above in Figure 8B. As with the models above, all of the dependent variables were transformed (arcsin of the square root) to meet the assumptions of linear mixed-effects models.

**FIGURE 11:**
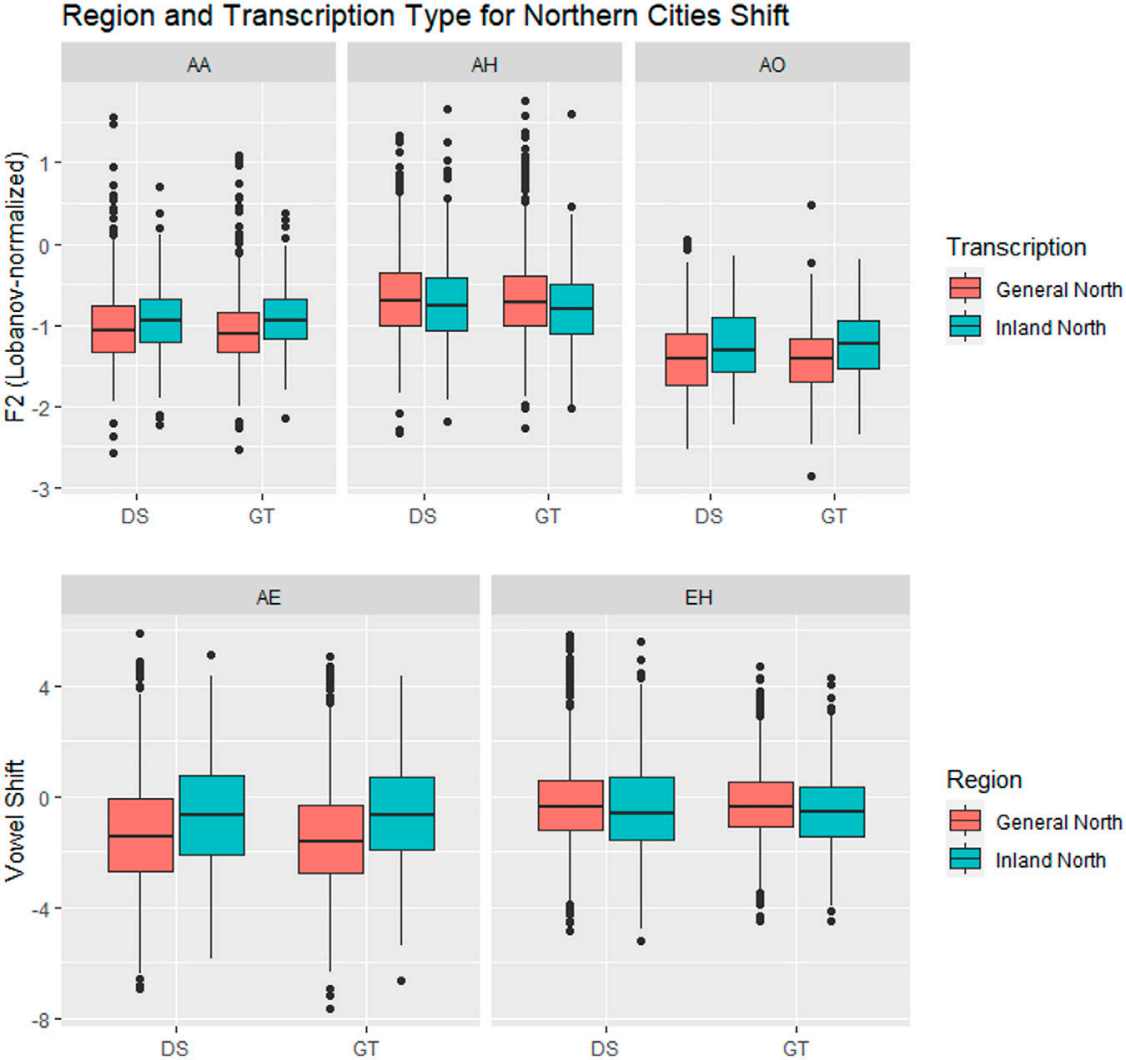
Vowels in the Northern Cities Shift, by Region (General North versus Inland North) and transcription type. In three of the vowels (AE, AA, AO) there are significant differences between General North and Inland North measurements.

The results for Region and Transcription type are shown in Figure 11 and Table 4A. For four out of five vowels, the behaviour of DeepSpeech transcribed vowels is similar to that of the vowels in the ground truth transcriptions. There is one vowel, stage 1 AE, where both ground truth and DeepSpeech found significant differences between General and Inland North vowels: ground truth shows a difference of ΔRegion_GT_ = 0.83 and DeepSpeech shows a significantly smaller but non-zero difference of ΔRegion_GT_ = 0.65 [β_GT:InlandNorth_ = −0.02 ± 0.008, t (256) = −2.0, *p* < 0.05]. Even though the distance between General and Inland North is smaller for DeepSpeech, it is significantly different from zero, as shown by the post-hoc analysis (z = 4.8, *p* < 0.0001). On the other hand, there are three vowels (stage 3 AO and stage 4 EH and stage 5 AH) where neither DeepSpeech nor the ground truth data could see significant differences between General and Inland vowels. For example, EH showed raising differences of ΔRegion_GT_ = 0.21 and ΔRegion_GT_ = 0.18, but neither of these were significantly different from zero (*p* = 0.88 and *p* = 1.0 respectively). In summary, for these four vowels (AE, AO, EH, and AH) both systems show similar patterns for both the General and Inland North speakers.

**TABLE 4:** LMER models for the Northern Cities Shift. The dependent variable is F2 for thought (AO), lot (AA) and strut (AH), and F2-2xF1 for trap (AE) and dress (EH). *R* ^2^ shows the marginal and conditional coefficients. Deltas show the difference between the mean position of the Southern vowel and the mean position of the Northern vowel for each transcription type: AA shows divergence in GT/DS results; for the other vowels, either both models detect a difference between Inland and General North or they do not.

A. Results for region and transcription type; *R* ^2^ for entire model						
**Vowels**	**Region by Transcription**	**Region**	**Transcription**	**Post-hoc ΔRegion_GT_**	**Post-hoc ΔRegion_DS_**	***R* ^2^**
AA (*n* = 1901)	*p* = 0.08	*p* = 0.29	*p* = 0.50	Δ = 0.15 t (94) = 2.9, *p* < 0.05	Δ = 0.07 *p* = 0.76	0.01
						0.72
AE (*n* = 3433)	β_GT:InlandNorth_ = −0.02 ± 0.01 t (256) = −2.0, *p* < 0.05	β_InlandNorth_ = −0.05 ± 0.01 t (312) = −4.8, *p* < 0.0001	*p* = 0.47	Δ = 0.83 z = 6.8, *p* < 0.0001	Δ = 0.65 z = 4.8, *p* < 0.0001	0.27
						0.67
AH (*n* = 3388)	*p* = 0.13	*p* = 0.24	β_GT_ = −0.02 ± 0.007 t (2703) = −3.0, *p* < 0.005	Δ = 0.12 *p* = 0.07	Δ = 0.06 *p* = 0.64	0.02
						0.54
AO (*n* = 1049)	*p* = 0.72	*p* = 0.12	*p* = 0.15	Δ = 0.18 *p* = 0.58	Δ = 0.16 *p* = 0.46	0.03
						0.74
EH (*n* = 4016)	*p* = 0.31	*p* = 0.98	*p* = 0.12	Δ = 0.21 *p* = 0.88	Δ = 0.18 *p* = 1.0	0.03
						0.48
**B. Results for other social and linguistic variables in the model**						
**Vowels**	**Year of birth**	**Gender**	**Following environment (Nasal vs. voiced obstruent)**		**Following environment (Nasal versus voiceless obstruent)**	
AA (*n* = 1901)	β = −0.01 ± 0.004, t (137) = −2.5, *p* < 0.05	*p* = 0.68	*p* = 0.68		*p* = 0.41	
AE (*n* = 3433)	β = 0.02 ± 0.004, t (192) = −3.9, *p* < 0.0005	β_male_ = 0.02 ± 0.007, t (191) = 2.5, *p* < 0.05	β_Nas/+VoicedObs_ = −0.12 ± 0.01, t (219) = −8.3, *p* < 0.00001		β_Nas/-VoicedObs_ = −0.17 ± 0.01, t (359) = −14.9, *p* < 0.00001	
AH (*n* = 3388)	*p* = 0.08	*p* = 0.25	*p* = 0.80		*p* = 0.06	
AO (*n* = 1049)	*p* = 0.17	*p* = 0.20	*p* = 0.15		*p* = 0.58	
EH (*n* = 4016)	β = 0.01 ± 0.004, t (172) = −3.8, *p* < 0.0005	β_male_ = 0.02 ± 0.008, t (172) = 3.1, *p* < 0.005	*p* = 0.42		β_Nas/-VoicedObs_ = −0.04 ± 0.01, t (310) = −3.4, *p* < 0.001	

There is one vowel, stage 2 AA, where there is a significant General/Inland North difference in the ground truth data [ΔRegion_GT_ = 0.15, t (94) = 2.9, *p* < 0.05], but there was no significant difference in the DeepSpeech transcriptions (ΔRegion_GT_ = 0.07, *p* = 0.76). This is the one vowel where the two transcription systems diverge. It should be noted that the ground truth data showed differences in the stage 1 and 2 vowels, the ones where the change is presumably more advanced, and it failed to show differences in the subsequent stages 3 through 5. In general, these results show that for most vowels the DeepSpeech data and the manual transcription are similar in how they portray the Northern Cities Shift: Either both of them show the vowel shifts (as is the case for AE) or both of them fail to do so (as is the case for AH, AO, and EH). Only in one of the vowels (AA) was the DeepSpeech data less able to detect the shift.

Like in the case of the Southern Vowel Shift, there are well established Northern Cities Shift linguistic patterns that are visible in the data (Labov et al., 2006). Three out of five vowels show significant differences due to the age of the speakers (stage 1 AE, stage 2 AA, and stage 4 EH): In AE and EH, younger speakers have greater shift; in AA, older speakers have greater shift. Two of the vowels show differences due to gender (stage 1 AE and stage 4 EH): male speakers show greater shift than female speakers. Finally, two of the vowels show differences in the vowel position due to the sounds that follow them (stage 1 AE and stage 4 EH). Also, like in the Southern Vowel Shift data, the *R* ^2^ correlation coefficients in Table 4 show that the random variable structure (individual speaker and word variation) explains significant amounts of the variation found in the dataset: the correlation from the fixed variables ranges from *R* ^2^ = 0.01 to *R* ^2^ = 0.27, while the *R* ^2^ for the data including the random variable greatly increases (up to *R* ^2^ = 0.74 in the case of AO). The summary of the variance explained by each of the random variables is in the Supplementary Appendix.

In summary, this section provides further evidence that the DeepSpeech data can detect phonetic differences in a manner similar to human-transcribed data. The majority of the vowels involved in the Northern Cities Shift, four out of five, showed a similar behavior in both transcriptions. This matches the pattern we saw with the vowels in the Southern Vowel Shift, where eight out of nine vowels also behaved in a similar manner across both transcription methods.

## Conclusion

Manual transcription has long been a bottleneck in sociophonetic vowel research. In this paper we have used a large audio dataset of North American English (352 speakers in the International Accents of English Archive) to show that automated speech recognition algorithms (ASR) can be an effective way to perform sociophonetic work for some types of large-scale research questions. We find that end-to-end deep learning based speech recognition algorithms (e.g., DeepSpeech) provide transcriptions that are closer to hand-transcribed data than in prior sociophonetic work. Furthermore, we find that sociophonetic analyses based on these fully automated transcription methods are effective in showing classic sociophonetic patterns of North American English, such as the Southern Vowel Shift (SVS) and the Northern Cities Shift (NCS), with significantly less effort and time invested than manual transcription approaches.

While these DeepSpeech transcriptions are not perfect, we find that they can still be used to gain valuable sociophonetic information. The sociophonetic results derived from the DeepSpeech transcriptions show that the Southern Vowel Shift and the Northern Cities Shift can in fact be graphically observed with these completely automated methods, even as the fine-grained statistical analyses show the ways in which DeepSpeech still lacks the higher degree of precision that can be obtained in analyses based on ground truth (manual) transcription. It also shows that there have been gains in areas relevant to sociolinguistic research, such as the improved transcription of female speech relative to previous ASR methods, as well as the similarity in transcription quality between the standard dialect of North American English and other regional dialects like Southern English.

Much future work remains in order to automatize sociophonetic transcriptions. For example, work needs to be done on whether the method presented here would also detect consonantal sociophonetic variation, given that consonants might not be recognized as reliably as vowels due to their shorter duration. Work also needs to be done on whether this method can be applied to other regional dialects and ethnolects. It is known that English dialects outside of North America, such as New Zealand and Scottish English, are transcribed less accurately (Tatman, 2017), so this method might not be able to detect vowel differences within those dialects. Also, as mentioned above, 79% of the sample was white, and therefore these statistical models might not accurately reflect how the method would perform when transcribing North American ethnolects like Black English, which are not well represented in ASR training corpora (Koenecke et al., 2020). We expect the accuracy of the ASR to be highly variable depending on the types of training input that it received and this could limit the broader application of this method to more diverse datasets. Finally, semi-automated methods like forced alignment have been fruitfully used to phonetics and sociophonetics in languages with extremely small datasets like Yoloxóchitl Mixtec (DiCanio et al., 2013), so there is the potential to apply speech recognition to describe linguistic variation in those languages as well.

Our results suggest that the technology for completely automated methods in vowel sociophonetics is closer to the point where such methods can reliably generate results that are similar to, if not quite the same as, results obtained by the painstaking process of manual transcription. After all, in any scientific endeavor, there is a tradeoff between accuracy and speed, and each research project can determine what type of approach is appropriate. For some sociolinguistic applications and large-scale research questions, such as “big data” analyses of huge sets of audio recordings, it may now be possible to use completely automated methods for reasonably reliable results.

## Data Availability

Publicly available datasets were analyzed in this study. This data can be found here: https://www.dialectsarchive.com. The code to process the data is available at: https://github.com/rolandocoto/darla-sociophonetics-2021.

## Footnotes

2
https://github.com/mozilla/DeepSpeech/releases/tag/v0.9.3

3 Recordings and associated text files are copyright and used by special arrangement with the International Dialects of English Archive at https://www.dialectsarchive.com

4 CER is defined as (substitutions + deletions + insertions)/length of source. In the case of BAT/CAT, only one letter is substituted in a string of length three, and therefore CER = 1/3 = 0.33. If both the source and the target transcriptions are identical (e.g., BAT/BAT), then the CER is zero. If the transcription is wrong but has the same length, then CER = 1 (e.g., BAT/DOG). If the transcription is longer than the original, then the CER can be greater than one (e.g., cat/foxes, CER = 1.66)

5 The vowels in this paper are transcribed using the ARPABET system for American English, as found in the CMU dictionary and in FAVE output spreadsheets: IY = FLEECE, UW = GOOSE, IH = KIT, EY = FACE, EH = DRESS, AE = TRAP, AW = MOUTH, AY = PRICE, AA = LOT, AO = THOUGHT, AH = STRUT, OW = GOAT, ER = NURSE, UH = FOOT, OY = CHOICE, and AHR = START

6 Most speakers in the IDEA dataset have a specific Year of Birth listed. But for a handful of speakers, the Year of Birth is just given as a decade, such as “1950s.” For such speakers, we simply estimated the Year of Birth at the middle of the decade, i.e., 1955
