# Supplementary material for: Advances in Completely Automated Vowel Analysis for Sociophonetics: Using End-to-End Speech Recognition Systems With DARLA
Source: Front Artif Intell. 2021 Sep 24;4:662097. doi: 10.3389/frai.2021.662097 (PMC8498339; doi:10.3389/frai.2021.662097)
Supplement: Supplementary file 1 [file DataSheet1.docx]

Appendix

1. Formulas for linear mixed-effects modelling

Dependent and fixed variables:

{F2 / vowelShifting } ~ region * transcriptionType + yearOfBirth + gender +

followingEnvironment

Random effects, General North / South:

AE: (1 + yearOfBirth + followingEnvironment | speaker) +

(1 + region + transcriptionType + followingEnvironment |word)

AW: (1 + region + followingEnvironment | speaker) +

(1 + region + transcriptionType + followingEnvironment |word)

EY: (1 + region + transcriptionType + yearOfBirth + followingEnvironment | speaker) +

(1 + region + transcriptionType + followingEnvironment |word)

EH: (1 + region + transcriptionType + gender + followingEnvironment | speaker) +

(1 + region * transcriptionType | word)

IY: (1 + transcriptionType + gender + followingEnvironment | speaker) +

(1 + region * transcriptionType + followingEnvironment | word)

IH: (1 + transcriptionType + followingEnvironment | speaker) +

(1 + region + transcriptionType + followingEnvironment | word)

OW: (1 + transcriptionType + followingEnvironment | speaker) +

(1 + region + transcriptionType | word)

UW: (1 + followingEnvironment | speaker) +

(1 + region |word)

Random effects, General North / Inland North

AA: (1 + transcriptionType + gender + followingEnvironment | speaker) +

(1 + followingEnvironment |word)

AE: (1 + followingEnvironment | speaker) +

(1 + followingEnvironment |word)

AH: (1+ gender + followingEnvironment | speaker) +

(1 + region +followingEnvironment |word)

AO: (1 + followingEnvironment | speaker) +

(1 + region + followingEnvironment |word)

EH: (1 + region + gender + followingEnvironment | speaker) +

(1 + followingEnvironment |word)

1. Random Effect Structure for Southern Vowel Linear Mixed-Effects Models

| SVS Vowels | By word | | By speaker | | Residual |
| --- | --- | --- | --- | --- | --- |
| AE (n=5198) | Intercept  Region_South_  Transcription_GT_  FolEnv_+VoicedObs_  FolEnv_-VoicedObs_ | 0.010 ± 0.101  0.004 ± 0.065  0.0008 ± 0.029  0.006 ± 0.074  0.007 ± 0.085 | Intercept  YearOfBirth  FolEnv_+VoicedObs_  FolEnv_-VoicedObs_ | 0.006 ± 0.078  0.0006 ± 0.024  0.012 ± 0.111  0.011 ± 0.105 | 0.0076 ± 0.087 |
| AW (n=3116) | Intercept  Region_South_  Transcription_GT_  FolEnv_+VoicedObs_  FolEnv_-VoicedObs_ | 0.003 ± 0.051  0.0005 ± 0.022  0.003 ± 0.057  0.006 ± 0.079  0.007 ± 0.083 | Intercept  Region_South_  FolEnv_+VoicedObs_  FolEnv_-VoicedObs_ | 0.004 ± 0.064  0.007 ± 0.081  0.004 ± 0.061  0.003 ± 0.058 | 0.008 ± 0.091 |
| EY (n=5730) | Intercept  Region_South_  Transcription_GT_  FolEnv_+VoicedObs_  FolEnv_-VoicedObs_ | 0.007 ± 0.084  0.002 ± 0.046  0.0005 ± 0.023  0.007 ± 0.085  0.004 ± 0.064 | Intercept  Region_South_  Transcription_GT_  YearOfBirth  FolEnv_+VoicedObs_  FolEnv_-VoicedObs_ | 0.008 ± 0.091  0.007 ± 0.083  0.0005 ± 0.023  0.0003 ± 0.083  0.008 ± 0.092  0.007 ± 0.086 | 0.009 ± 0.096 |
| EH (n=5930) | Intercept  Region_South_:Transcription_GT_  Region_South_  Transcription_GT_ | 0.009 ± 0.095  0.003 ± 0.055  0.011 ± 0.105  0.001 ± 0.033 | Intercept:  Region_South_  Transcription_GT_  Gender_male_  FolEnv_+VoicedObs_  FolEnv_-VoicedObs_ | 0.006 ± 0.075  0.005 ± 0.069  0.0004 ± 0.021  0.004 ± 0.060  0.004 ± 0.066  0.005 ± 0.072 | 0.011 ± 0.104 |
| IY (n=4442) | Intercept  Region_South_:Transcription_GT_  Region_South_  Transcription_GT_  FolEnv_+VoicedObs_  FolEnv_-VoicedObs_ | 0.015 ± 0.124  0.005 ± 0.072  0.009 ± 0.095  0.0007 ± 0.027  0.004 ± 0.064  0.006 ± 0.077 | Intercept  Transcription_GT_  Gender_male_  FolEnv_+VoicedObs_  FolEnv_-VoicedObs_ | 0.008 ± 0.090  0.0006 ± 0.025  0.004 ± 0.061  0.004 ± 0.063  0.005 ± 0.070 | 0.008 ± 0.088 |
| IH (n=5961) | Intercept  Region_South_  Transcription_GT_  FolEnv_+VoicedObs_  FolEnv_-VoicedObs_ | 0.013 ± 0.110  0.004 ± 0.060  0.0004 ± 0.020  0.028 ± 0.168  0.002 ± 0.046 | Intercept:  Transcription_GT_  FolEnv_+VoicedObs_  FolEnv_-VoicedObs_ | 0.003 ± 0.059  0.0001 ± 0.012  0.004 ± 0.066  0.004 ± 0.067 | 0.007 ± 0.085 |
| OW (n=4611) | Intercept  Region_South_  Transcription_GT_ | 0.005 ± 0.071  0.0008 ± 0.028  0.0001 ± 0.011 | Intercept:  Transcription_GT_  FolEnv_+VoicedObs_  FolEnv_-VoicedObs_ | 0.004 ± 0.061  0.0004 ± 0.021  0.002 ± 0.047  0.002 ± 0.043 | 0.010 ± 0.098 |
| UW (n=2786) | Intercept  Region_South_ | 0.009 ± 0.097  0.002 ± 0.045 | Intercept  FolEnv_+VoicedObs_  FolEnv_-VoicedObs_ | 0.007 ± 0.083  0.003 ± 0.059  0.005 ± 0.069 | 0.010 ± 0.098 |

1. Random Effect Structure for Inland North Vowel Linear Mixed-Effects Models

| NCS Vowels | By word | | By speaker | | Residual |
| --- | --- | --- | --- | --- | --- |
| AA (n=1901) | Intercept  FolEnv_+VoicedObs_  FolEnv_-VoicedObs_ | 0.013 ± 0.113  0.025 ± 0.160  0.028 ± 0.169 | Intercept  Region_South_  Transcription_GT_  Gender_male_  FolEnv_+VoicedObs_  FolEnv_-VoicedObs_ | 0.003 ± 0.052  0.004 ± 0.061  0.001 ± 0.034  0.004 ± 0.060  0.006 ± 0.075  0.002 ± 0.048 | 0.005 ± 0.073 |
| AE (n=3433) | Intercept  FolEnv_+VoicedObs_  FolEnv_-VoicedObs_ | 0.009 ± 0.094  0.004 ± 0.064  0.003 ± 0.057 | Intercept  FolEnv_+VoicedObs_  FolEnv_-VoicedObs_ | 0.007 ± 0.084  0.015 ± 0.122  0.011 ± 0.105 | 0.009 ± 0.092 |
| AH (n=3388) | Intercept  Transcription_GT_ | 0.009 ± 0.096  0.0008 ± 0.029 | Intercept:  Gender_male_  FolEnv_+VoicedObs_  FolEnv_-VoicedObs_ | 0.002 ± 0.039  0.005 ± 0.069  0.002 ± 0.040  0.003 ± 0.058 | 0.009 ± 0.097 |
| AO (n=1049) | Intercept:  Region_South_  FolEnv_+VoicedObs_  FolEnv_-VoicedObs_ | 0.012 ± 0.107  0.004 ± 0.061  0.031 ± 0.177  0.039 ± 0.197 | Intercept:  FolEnv_+VoicedObs_  FolEnv_-VoicedObs_ | 0.010 ± 0.102  0.014 ± 0.117  0.016 ± 0.126 | 0.006 ± 0.076 |
| EH (n=4016) | Intercept:  FolEnv_+VoicedObs_  FolEnv_-VoicedObs_ | 0.008 ± 0.089  0.018 ± 0.136  0.006 ± 0.076 | Intercept:  Region_South_  Gender_male_  FolEnv_+VoicedObs_  FolEnv_-VoicedObs_ | 0.007 ± 0.082  0.003 ± 0.056  0.005 ± 0.072  0.004 ± 0.066  0.005 ± 0.069 | 0.011 ± 0.106 |
